# Carbon Dioxide Laser Microsurgical Median Glossotomy for Resection of Lingual Dermoid Cysts

**DOI:** 10.3389/fsurg.2016.00042

**Published:** 2016-07-25

**Authors:** Kristien Corvers, Greet Hens, Jeroen Meulemans, Pierre Delaere, Robert Hermans, Vincent Vander Poorten

**Affiliations:** ^1^Otorhinolaryngology, Head and Neck Surgery, University Hospitals Leuven, Leuven, Belgium; ^2^Section Head and Neck Oncology, KU Leuven Department of Oncology, Leuven, Belgium; ^3^Radiology, University Hospitals Leuven, Leuven, Belgium; ^4^KU Leuven Department of Imaging and Pathology, Leuven, Belgium

**Keywords:** lingual dermoid, CO_2_ laser, median glossotomy

## Abstract

Dermoid cysts are epithelial-lined cavities with skin adnexae in the capsule. Only 7% is present in the head and neck. Between 2004 and 2013, four patients with a lingual dermoid cyst underwent a microsurgical carbon dioxide laser resection *via* a median sagittal glossotomy approach. This approach is an elegant technique combining superior visualization, hemostasis, and little postoperative edema with good wound healing, allowing for perfect function preservation of the tongue.

## Introduction

Dermoid cysts are squamous epithelial-lined cavities with a variable number of skin adnexae in the capsule (1). Only 7% of dermoid cysts are present in the head and neck, most commonly in the periorbital region (50%), followed by the floor of the mouth, submental, and submaxillary (23%), frontal and neck (14%), and nasal region (13%) (2).

Histological varieties include epidermoid cysts (lined with simple squamous epithelium and surrounding connective tissue without skin appendages), dermoid cysts (epithelial-lined cavity also containing dermal appendages, such as sebaceous and sweat glands, and hair follicles), and teratomas [epithelial-lined cavity that contains mesodermal end endodermal derivates, including muscle, cartilage, intestinal mucosa, and bone (1)].

These lesions are usually discovered at birth or during the first year of life. A second peak occurs during adolescence (3, 4). Males and females are equally affected (5). They present as a painless, slowly growing swelling, covered by normal mucosa, which enlarges until difficulties of articulation, mastication, and deglutition or airway compromise occur (6, 7). Infections can occur and recurrent infections can result in a sinus tract (8).

Regarding the etiology of these cysts, two theories of pathogenesis exist. The first hypothesis goes that, during the embryonic development, the anterior two-thirds of the tongue develop from fusion of three swellings of primitive mesenchyme: two lateral swellings (the lateral processes) and a median swelling (the tuberculum impar of His). During the 4th week of embryonic development, the two lateral processes migrate medially to fuse over the tuberculum impar forming the tongue corpus. During this fusion, entrapment and subsequent proliferation of epithelial debris is thought to result in the formation of a dermoid cyst. A second hypothesis states that epidermal and dermal cells trapped following trauma could result in the formation of an acquired dermoid cyst (9–11).

The differential diagnosis of a painless swelling in the tongue midline includes cystic hygroma, neurofibroma, hemangioma, lingual thyroid, teratoma, foregut duplication cyst, and dermoid cyst (10).

Magnetic resonance imaging (MRI) is the best modality for the diagnosis and follow-up of a lingual dermoid cyst. These cysts appear as sharply demarcated mass lesions, iso- or hypointense to muscle on T1-weighted images and hyperintense on T2-weighted images. The diagnosis can be suggested when a spontaneous T1-hyperintense fat component is seen on MRI. Focal areas of low signal intensity may be seen if calcifications are present (12).

## Case Presentations

Between 2004 and 2013, four patients with a lingual dermoid cyst underwent a microsurgical carbon dioxide (CO_2_) laser resection of a cyst via a median sagittal glossotomy approach in our department by a senior surgeon (Vincent Vander Poorten).

### Case 1

**Figure 1:**
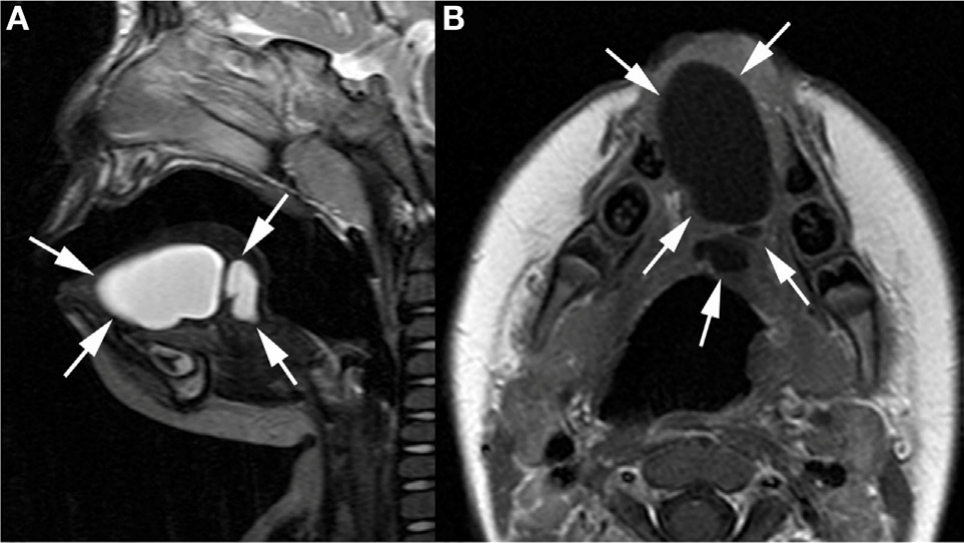
**Sagittal T2-weighted (A) and axial gadolinium-enhanced T1-weighted image (B) show well-defined cystic lesion (arrows) in the midline of the oral tongue, septated in its posterior part**.

A 7-week-old boy presented with a cystic swelling of the left tongue tip and inferior side of the tongue. The cyst was discovered on ultrasound at 28 weeks of gestation. An immediate postnatal MRI revealed a well-defined midline structure in the anterior half of the tongue of 3.6 cm (Figure 1). Because the cyst was asymptomatic, surgery was delayed until the age of 1 year.

### Case 2

**Figure 2:**
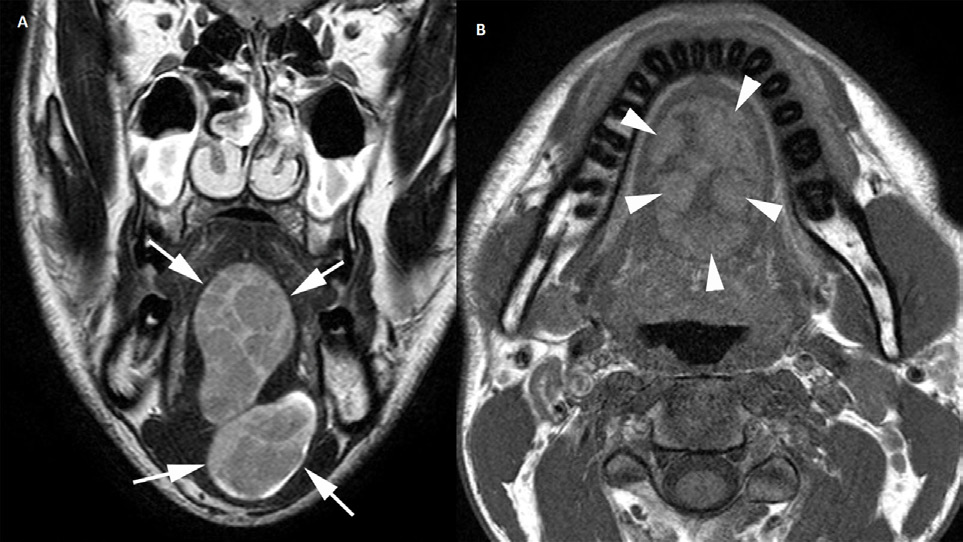
**Coronal T2-weighted image (A) depicts well-demarcated, bilobular heterogeneous mass lesion (arrows) in the midline of the tongue, extending in the floor of the mouth. The plain T1-weighted image (B) shows spontaneous hyperintense areas (arrowheads) within the lesion, indicating the presence of fat**.

A 2-year-old boy was referred to our department for macroglossia. Clinical examination showed a cystic midline protrusion of the tongue tip and corpus and a tongue ankylosis. MRI confirmed a well-demarcated cyst of 4.6 cm (Figure 2). At the age of two, two connected cysts were resected. A lingual frenulotomy was performed.

### Case 3

A 14-month-old girl was referred to us with an infection of the floor of the mouth following attempted needle aspiration of a suspected ranula 1 week earlier, not ameliorating under Amoxicillin–Clavulanic acid. The swelling had been noticed since the age of 2 months. The infection cooled down after partial needle aspiration of the liquid content, relieving pressure and facilitating penetration of antibiotic treatment with Piperacillin/tazobactam (Tazocin^®^) and metronidazole (Flagyl^®^). After remaining asymptomatic for 7 months, the swelling recurred. A prophylactic antibiotic treatment with Amoxicillin was started and the operation date was accelerated.

### Case 4

A 29-year-old man consulted us with a slowly progressive swelling, medially in the floor of the mouth, since 10 years. Clinical examination showed a soft well-defined cyst medially in the flour of the mouth and descending submentally. MRI showed a 7-cm bilocular midline cystic lesion in the floor of the mouth.

## Surgical Technique

**Figure 3:**
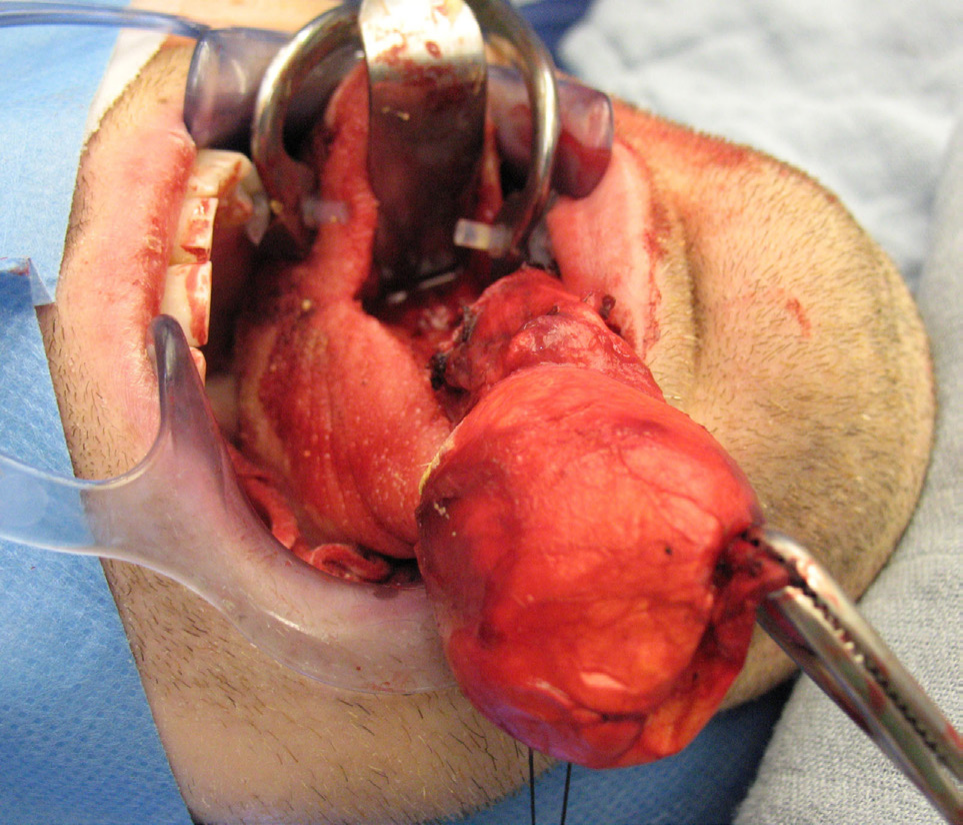
**Median sagittal glossotomy approach with progressive dissection of a big bilocular cystic lesion (case 4)**.

**Figure 4:**
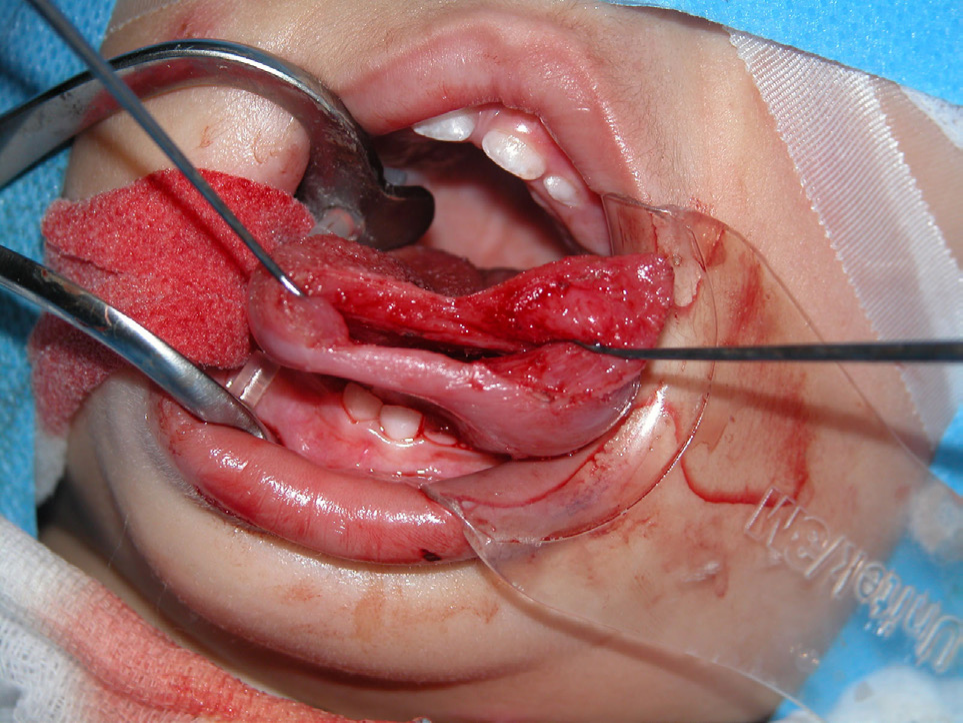
**Median sagittal glossotomy approach after resection of the cyst (case 2)**.

All four patients were operated under general anesthesia with nasotracheal intubation (see Video S1 in Supplementary Material). The operations were all performed by one senior surgeon (Vincent Vander Poorten). A retractor was placed in the mouth and on each side of the tongue two Vicryl^®^ suspension sutures were placed. Next, the dissection started using the CO_2_ laser (Acuspot712-Lumenis, Yokneam, Israel) mounted on a Zeiss operating microscope (Zeiss Opmi, Oberkochen, Germany) at a magnification of 12.5, using 1.5 watt in a superpulse mode. The mucosa was incised using the laser and the tongue musculature was cleaved on the median raphe using “sharp” laser dissection until visualization of the cyst, which was then further dissected by a combination of laser and blunt dissection, using a small mosquito-mounted dissecting swab (Figures 3 and 4). Following delivery of the cyst, the tongue musculature was reapproximated with interrupted 4.0 Vicryl^®^ sutures and the mucosa was closed using 5.0 Monocryl^®^. The operation time ranged from 110 to 165 min. Peroperatively all patients received corticosteroids (*methylprednisolon sodiumsuccinate* 1 mg/kg) and antibiotics (cefazoline 50 mg/kg).

Postoperative intubation and close monitoring in the intensive care unit (ICU) are advisable. Two patients were extubated the same day, the adult in the operation room, and one child by accident in the ICU. The two other children were extubated the next day. ICU observation ranged from 1 to 3 days.

In one child, a nasogastric tube was placed. In the adult, because of the large volume of the resected cyst, a drain was placed in the floor of the mouth. Both could be removed after 1 day.

Eating and drinking was allowed from the first postoperative day. One child with a preoperative infection only started eating at postoperative day 5. The other patients had no difficulties eating.

All patients received prophylactic antibiotics during 1 week: Amoxicillin or Amoxicillin–Clavulanic acid, two patients received corticosteroids for 1 day.

The patients were discharged home ranging from postoperative day 2 to postoperative day 6.

## Results

Four lingual dermoid cysts, all of substantial volume, were resected with CO_2_ laser via a median sagittal glossotomy approach.

In all patients, an iatrogenic perforation of the cyst occurred during dissection, but the cyst wall could further be preserved intact and removed completely.

The postoperative course was uneventful except for difficulties in eating in one patient and one loose suture that did not cause wound dehiscence. In all patients, the function (both motoric and sensory) of the tongue returned to normal in the immediate postoperative days. With a follow-up ranging from 2 to 11 years after the operation, there is no disease recurrence in any of the patients.

## Discussion

Treatment of a dermoid cyst consists of complete excision, aiming at preserving breathing and swallowing, adequate speech, taste, sensation, and normal oro-facial development (5, 13). Timing of the treatment varies. When possible, surgery is delayed in a child under 20 kg because of the greater anesthetic risk (4), knowing that this delay can lead to inflammatory complications requiring incision and drainage.

There are different intra- or extra-oral surgical options available. The approach chosen must provide appropriate access for complete excision and, at the same time, preserve the delicate innervation and motoric function of the tongue. For this reason, we favor the midline sagittal glossotomy approach. This approach has already been used for sublingual dermoid cysts (4), tumors of the posterior third of the tongue, and as part of a labiomandibular glossotomy for access to the clivus and craniovertebral junction of the upper cervical spine (14). Placing the incision in the tongue along the median raphe, an avascular plane, minimizes the blood loss as well as the risk of damage to the submandibular ducts and sublingual glands, while maximally sparing the tongue musculature and the sensible and motor innervation, that come in from laterally. Using two suspension sutures for retraction facilitates exposure.

The development of a bifid tongue when there is incomplete healing of the tip, resulting in articulation difficulties (5) and the development of a mucocoele because of trauma to the sublingual salivary gland (15) are theoretical complications, which we did not encounter.

The advantages of our approach are the superior binocular microscopic vision in combination with the precise cutting capacities and good hemostatic effect by sealing of small blood vessels by the CO_2_ laser, providing a dry surgical field, in the highly vascular tissue of the tongue. There is also minimal inflammation and postoperative edema because of sealing of the lymphatics as well as sterilization of the wound, reducing the risk of postoperative infections (16, 17). Precise cutting in the tongue is easier with the CO_2_ laser as compared to electrocautery and scalpel dissection because there are no muscle fasciculations (17). The CO_2_ laser also produces less postoperative pain than electrocautery or conventional scalpel procedures, but the mechanism underlying this observation is still unknown (18). In summary, this technique combines maximal preservation of the non-involved tongue musculature and neurovascular structures with prevention of soft tissue swelling.

In the literature, we found one report of a lingual dermoid cyst in a 4-month-old-girl excised with the CO_2_ laser using the median sagittal glossotomy approach, which was published after we already had used this technique in two patients, waiting for follow-up to accumulate. That patient experienced postoperative feeding difficulties for 48 h probably due to pain (19). Our patient with the preoperative infection also had feeding difficulties for 5 days, and the other patients did not experience problems.

## Conclusion

The resection of a tongue-dermoid cyst with CO_2_ laser via a median sagittal glossotomy approach is an elegant technique combining superior visualization, hemostasis, and little postoperative edema with good wound healing, allowing for perfect function preservation of the tongue.

## Author Contributions

KC collected the data and drafted the manuscript. GH, JM, and PD were involved in patient care and follow-up and critically revised the manuscript. RH was involved in patient care and follow-up, preparation of the imaging, and critically revised the manuscript. VP conceived the study, was involved in patient care, surgery, and follow-up, drafted the manuscript, and critically revised the manuscript.

## Conflict of Interest Statement

The authors declare that the research was conducted in the absence of any commercial or financial relationships that could be construed as a potential conflict of interest.
